# Class I histone deacetylase inhibition promotes CD8 T cell activation in ovarian cancer

**DOI:** 10.1002/cam4.3337

**Published:** 2020-12-24

**Authors:** Tyler R. McCaw, Nidhi Goel, Dewey J. Brooke, Ashwini A. Katre, Angelina I. Londoño, Haller J. Smith, Troy D. Randall, Rebecca C. Arend

**Affiliations:** ^1^ Division of Clinical Immunology and Rheumatology Department of Medicine University of Alabama at Birmingham Birmingham AL USA; ^2^ Division of Gynecology Oncology Department of Obstetrics and Gynecology University of Alabama at Birmingham Birmingham AL USA

**Keywords:** epigenetics, gynecological oncology, immunology, tumor‐infiltrating immune cells

## Abstract

**Objective:**

Patients with epithelial ovarian cancer (EOC) typically present with late‐stage disease, posing a significant challenge to treatment. Although taxane and platinum‐based chemotherapy plus surgical debulking are initially effective, EOC is marked by frequent recurrence with resistant disease. Immunotherapy represents an appealing treatment paradigm given the ability of immune cells to engage metastatic sites and impede recurrence; however, response rates to checkpoint blockade in ovarian cancer have been disappointing. Here, we tested whether class I HDAC inhibition can promote anti‐tumor T cell responses in a spontaneous and nonspontaneous murine model of EOC.

**Methods:**

We used the spontaneous Tg‐MISIIR‐Tag and nonspontaneous ID8 models of murine ovarian cancer to test this hypothesis. Whole tumor transcriptional changes were assessed using the nCounter PanCancer Mouse Immune Profiling Panel. Changes in select protein expression of regulatory and effector T cells were measured by flow cytometry.

**Results:**

We found that treatment with the class I HDAC inhibitor entinostat upregulated pathways and genes associated with CD8 T cell cytotoxic function, while downregulating myeloid derived suppressor cell chemoattractants. Suppressive capacity of regulatory T cells within tumors and associated ascites was significantly reduced, reversing the CD8‐Treg ratio.

**Conclusions:**

Our findings suggest class I HDAC inhibition can promote activation of intratumoral CD8 T cells, potentially by compromising suppressive networks within the EOC tumor microenvironment. In this manner, class I HDAC inhibition might render advanced‐stage EOC susceptible to immunotherapeutic treatment modalities.

## Graphical abstract

Expression of genes corresponding to CD8 T cell activation and effector function are upregulated following entinostat treatment. Class I HDAC inhibition also compromises the intratumoral suppressive network at multiple nodes in epithelial ovarian cancer. Accordingly, class I HDAC inhibition can be integrated into current treatment strategies to promote endogenous anti‐tumor immune responses and sensitize ovarian cancer to immunotherapy.

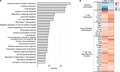

## 1 INTRODUCTION

Absence of reliable screening tools and nonspecific symptoms frequently delay patient presentation until epithelial ovarian cancer (EOC) has progressed to late‐stage disease. Taxane and platinum‐based chemotherapeutics plus surgical debulking are generally efficacious; however, EOC is marked by frequent recurrence with resistant disease, rendering subsequent therapeutic approaches iteratively less successful.^1^ Incorporation of anti‐vascular endothelial growth factor (anti‐VEGF) and poly‐ADP ribose polymerase (PARP) inhibitors has modestly improved progression‐free survival (PFS) in appropriately selected patients^2^, ^3^ but overall improvements in five‐year survival remain underwhelming.^4^ Accordingly, new approaches to the treatment of EOC are critically needed.

Since recognition that patients’ endogenous immune system can recognize and productively engage transformed cells, strategies aimed at immune potentiation, notably checkpoint blockade, have transformed the treatment landscape in several cancers.^5^ Infrequent responses to checkpoint blockade have thus far limited its application in EOC,^6^ but immunotherapy may yet represent a promising approach, as T cells receiving appropriate signals can disseminate widely to combat intra‐ and extra‐peritoneal disease and delimit potential for recurrence of resistant subpopulations through epitope spreading.^7^ Indeed, the presence of T cells within ovarian tumors correlates with significantly more favorable outcomes.^8^ Patient presentation at late stages, though, poses a notable challenge to immunotherapeutic approaches, as with chemotherapeutic ones. In late stages, EOC has progressed to escape phase, wherein disease has subverted cytotoxic CD8 T cells in favor of suppressive populations to create a hostile tumor microenvironment (TME).^9^

EOC is characterized by a uniquely suppressive TME largely maintained by tumor‐associated populations of regulatory T cells (Tregs) that suppress effector T cell cytotoxic functions.^10^, ^11^ Treg density and the CD8 T cell‐Treg ratio reflect degree of suppression, with lower ratios corresponding to poor overall survival.^12^ Suppressive populations of other lineages, as well as tumor cell‐intrinsic changes, further impair effective anti‐tumor immunity.^13^, ^14^ Therefore, stimulating productive tumor‐specific T cell responses may necessitate disruption of both tumor cell and suppressive cell programs.

Histone deacetylase (HDAC) inhibitors shift HDAC‐histone acetyl transferase equilibria in all cells, tumor and immune cell alike, to alter chromatin frameworks and consequent expression profiles. Initial development of these agents focused on their direct anti‐tumor effects, including induced differentiation, reduced viability, and impaired proliferation.^15^ However, HDAC inhibitors can also promote T cell‐mediated anti‐tumor immunity through direct (enhanced effector functions) and indirect (compromised functions of suppressive populations in the TME) mechanisms.^16^ Thus, HDAC inhibitors may be well‐suited immunotherapeutic adjuvants capable of promoting productive tumor‐specific T cell responses in EOC.

Using both spontaneous and non‐spontaneous murine models of EOC, we tested this idea to show how class I HDAC inhibition confers improved anti‐tumor immunity. We found that treatment of tumor‐bearing mice with entinostat (a class I inhibitor, ENT) upregulates expression profiles and phenotypic features associated with improved CD8 T cell effector functions while reducing Treg‐mediated suppression within the TME and surrounding ascites. These findings suggest class I HDAC inhibition could alleviate the suppression within the EOC TME, facilitating cytotoxic T cell responses and increasing response rates to other immunotherapeutic modalities.

## 2 METHODS

### 2.1 Cell culture

ID8 cells—a nonspontaneous model of murine epithelial ovarian cancer—were obtained from Dr Yancey Gillespie (University of Alabama at Birmingham). Cells were cultured in RPMI1640 (Mediatech, Inc) with 10% fetal bovine serum (FBS; Atlanta Biologicals, Flowery Branch). Cell cultures were passaged 3‐5 times from thaw prior to injection. Cultures at 60%‐90% confluency were passaged by dissociation with 0.05% trypsin, 0.53mM EDTA (Corning, Inc) incubation for 5 minutes, washed with sterile PBS (Mediatech, Inc), and transferred to fresh media.

### 2.2 Chemicals and reagents

>99% ENT was purchased from LC laboratories. Stock solutions were prepared by dissolving ENT in DMSO, stored at −20°C, and were not freeze‐thawed more than twice. Prior to injections, ENT stocks were diluted in vehicle: 15% DMSO (Sigma), 20% Kolliphor EL (Sigma), and 65% phosphate buffered saline (PBS).

### 2.3 Mouse models

All animal studies were approved by the University of Alabama at Birmingham, Birmingham, AL, US Institutional Animal Care and Use Committee (IACUC) and in accordance with the guidelines of the National Research Council (US) Committee for the Update of the Guide for the Care and Use of Laboratory Animals.

Following culture, 7 × 10^6^ ID8 cells were prepared in 200 uL sterile PBS and injected into the peritoneal cavity of syngeneic C57BL/6 mice. Daily vehicle or 20 mg/kg ENT was started on day 21 post tumor cell injection and was administered by intraperitoneal injection. Mice were sacrificed and omental tumors and ascites fluid were harvested on day 31.

The Tg‐MISIIR‐Tag strain represents a spontaneous model of serous ovarian cancer driven by SV40 transforming region expression controlled by the Mullerian Inhibitory Substance type II Receptor (MISIIR) promoter region on a C57BL/6 background. Mice were obtained from Dr Denise Connolly (Fox Chase Cancer Center).^17^ These mice spontaneously develop ovarian tumors that metastasize to the peritoneal cavity by approximately week 10 of life. Ten‐week‐old mice were randomized to vehicle or 5 mg/kg ENT treatment, and the corresponding treatment was given by intraperitoneal injection on 5 consecutive days with a two day drug holiday each week until harvest at 7 or 10 weeks. A lower dose of ENT was used given the protracted treatment duration.

### 2.4 NanoString nCounter mRNA and pathway analysis

At pre‐specified time points, omental tumors were harvested and whole‐tumor mRNA extracted using TRIzol Plus RNA Purification Kit (Life Technologies Corp.). Tumors were mechanically homogenized in 500 uL of TRIzol reagent added and passaged 5‐10 times through an 18 gauge syringe. The remainder of purification was performed as per the manufacturer's instructions. RNA quantification was performed using the DeNovix DS‐11 Spectrophotometer (DeNovix, Inc). Samples were processed on the NanoString nCounter Flex System per manufacturer instructions using the nCounter PanCancer Mouse Immune Profiling panel (NanoString Technologies, Inc), a gene set interrogating 750 cancer‐related genes alongside 20 internal reference controls (full gene list and controls available on manufacturer's website). nSolver 2.6 software (NanoString Technologies, Inc) was used for data analysis. Pathway analysis was performed using Metascape, a web‐based portal for gene list annotation.^18^ Here, genes with an absolute value fold change greater than or equal to 2 at the week 10 timepoint were collated and entered into the platform, M. musculus selected as input and analysis species, and available express analysis conducted. Fold change represents change in ENT‐treated samples vs vehicle‐treated.

### 2.5 Flow cytometry

Harvested tumors were mechanically dissociated by dicing and incubated in RPMI1640 media supplemented with 5% FBS, collagenase (c7657, Sigma), and DNase (d5025, Sigma) in a shaker incubator for 30 minutes at 37°C and 200 rpm. Cells were then passed through 70 um nylon filter (Corning, Inc) and washed with PBS to create single‐cell suspensions.

For staining, cells were resuspended in PBS with 2% donor calf serum and 10 μg/mL FcBlock (2.4G2, BioXCell). For surface staining, cells were incubated in antibody‐containing solution for 30 minutes at 4°C in the dark. For intracellular staining, cells were first fixed using the eBioscience FoxP3 Fixation Kit (ThermoFisher Scientific) for 20 minutes at room temperature. Fixed cells were permeabilized and intracellular proteins stained per manufacturer instructions for at least 45 minutes at 4°C in the dark. Antibodies against CD3 (17A2), Eomes (Dan11mag), and FoxP3 (FJK‐16s) were obtained from eBioscience. Antibodies against CD4 (GK1.5) and Tbet (4B10) were obtained from BioLegend. Antibodies against CD8 (53‐6.7) and CD25 (PC61) were obtained from BD Bioscience. LIVE/DEAD fixable dyes were obtained from Life Technologies. Samples were run on a BDFACS Canto II system (BD Biosciences) and the data was analyzed using FlowJo software version 9.9. Tregs were identified as live, CD3+, CD4+, CD25+, FoxP3+ cells, and CD8 T cells gated on live, CD3+, CD8+. Using a common gating strategy through CD3+, CD8‐Treg ratios were then calculated by dividing cumulative frequencies.

### 2.6 Statistical analysis

Determination of statistical significance between measured values in each cohort was calculated using independent two‐tailed *t* tests, assuming an alpha of 0.05. All statistical analyses were conducted using GraphPad Prism (version 7.0, GraphPad Software). Statistical significance of pathway analysis determined by Metascape.^18^

## 3 RESULTS

### 3.1 Class I HDAC inhibition upregulates effector T cell signatures in ovarian tumors

**FIGURE 1:**
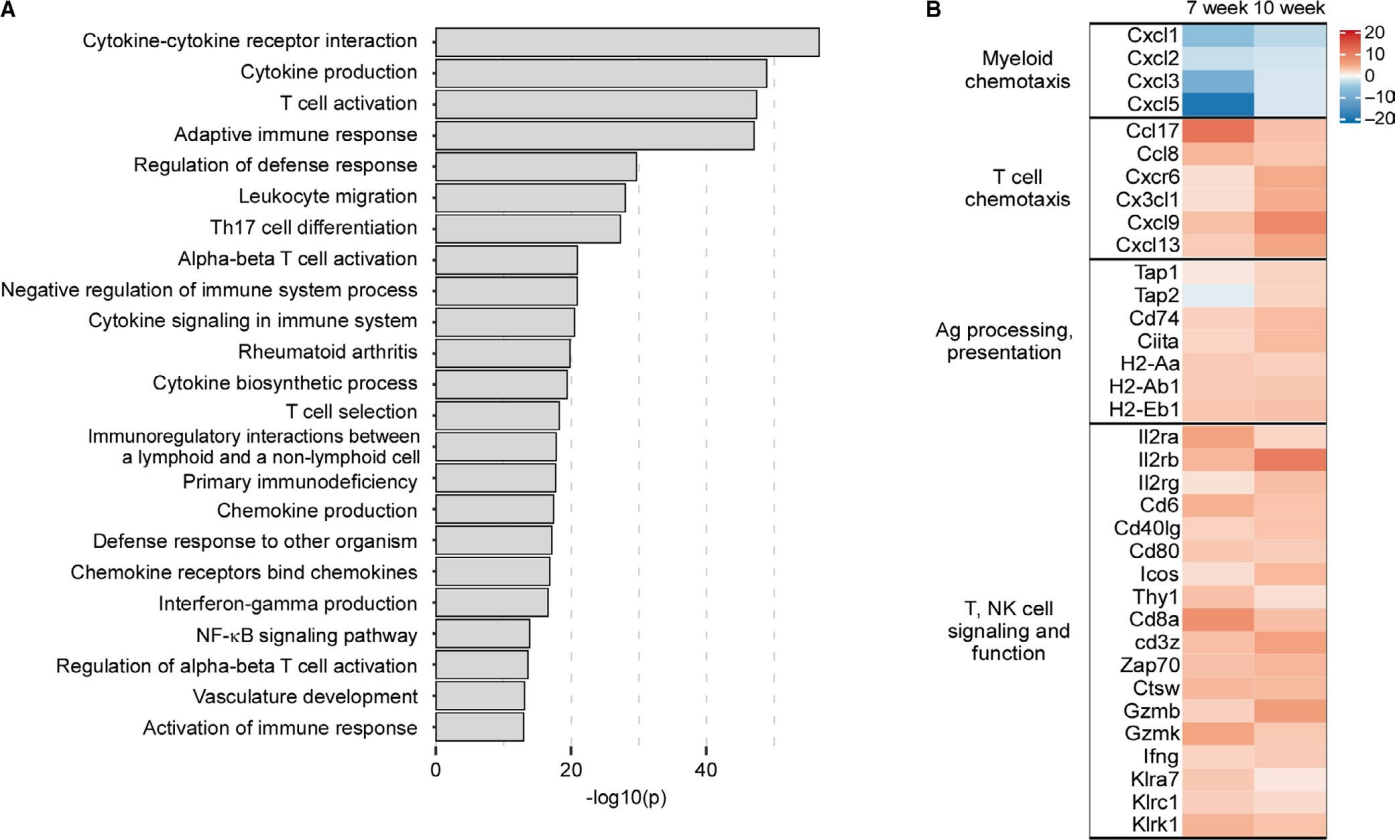
ENT promotes anti‐tumor T cell function in a spontaneous model of murine ovarian cancer. MISIIR mice at 10 wks of life, a time consistent with the development of gross ovarian tumors, were treated with vehicle or ENT for an additional 7 or 10 wks. Tumors were harvested and whole tumor lysate subjected to NanoString analysis. A, Pathway analysis of differentially expressed genes following 10 wks of treatment, top most differentially expressed pathways shown. B. Heatmap of specific gene expression after 7 and 10 wks of treatment

HDAC inhibitors, particularly class I, can upregulate expression of surface proteins involved in T cell‐mediated recognition and killing of tumor cells.^16^ To test whether these changes in tumor expression profiles improved T cell function within spontaneously arising ovarian cancer, MISIIR mice were treated with vehicle or ENT for 7 or 10 weeks, tumors harvested, and changes in gene expression of whole tumor lysate measured. Pathway analysis revealed the top 20 most differentially expressed pathways following 10 weeks of ENT treatment largely reflected enhanced T cell functionality, including chemotaxis, T cell receptor (TCR) and costimulatory signaling, and production of effector cytokines (Figure 1A), suggesting an increased ability of T cells to traffic to the tumor mass and effectively engage tumor cells. The 20 most upregulated genes were similarly enriched for those participating in T cell signaling and activation (Table S1). Accordingly, we found an increased expression of *ccl8*, *cxcr6*, *cx3cl*, *cxcl9*, genes involved in T cell recruitment to the TME (Figure 1B). Once in the tumor mass, CD8 T cells must effectively engage tumor cells. Indeed, genes involved in T cell costimulation (*il2ra*, *il2rb*, *il2rg*, *cd40lg*, *icos*, *cd80*), activation via signaling through the TCR (*cd8a*, *cd3z*, *zap70*, *ctsw*), and tumor killing (*gzmb*, *gzmk*, *ifng*, *klrc1*, *klrk1*) were consistently upregulated after 7 and 10 weeks of treatment. Increased functionality could be due to direct differences in CD8 T cell gene expression incurred by ENT treatment or through indirect effects, like increasing immunogenicity of tumor cells or decreasing function of suppressive populations within the TME. ENT treatment led to increased expression of *ciita*, *tap1*, *tap2*, *cd74*, and major histocompatibility genes—involved in antigen processing and presentation—suggesting an increased ability to T cells to engage tumor cells, consistent with previous reports.^16^ Importantly, expression of canonical T cell effector genes and those participating in antigen presentation are similarly increased in the nonspontaneous ID8 model following ENT treatment.^19^ Genes associated with myeloid chemotaxis (*cxcl1, cxcl2, cxcl3, and cxcl5*) were notably decreased by ENT suggesting reduced accumulation of suppressive myeloid populations within the TME. Collectively, these data indicate that daily treatment with ENT can promote CD8 T cell trafficking to and cytotoxic function in ovarian tumors, while potentially impairing suppressive populations therein.

### 3.2 Class I HDAC inhibition impedes Treg‐mediated suppression

**FIGURE 2:**
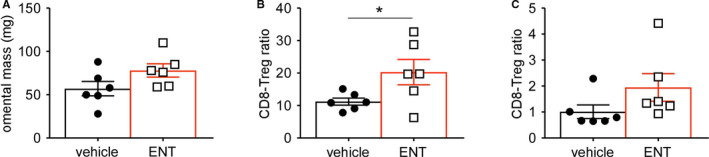
Class I HDAC inhibition reverses CD8‐Treg ratios in tumor and ascites. ID8 cells were injected into the peritoneal cavity of C57BL/6 mice, daily treatment with vehicle or ENT started on day 21, and omental tumors harvested after 10 d of treatment on day 31. Populations within tumors and ascites were assessed by flow cytometry. A, Mass of omentum at time of harvest. B, CD8‐Treg ratio in peritoneal ascites. C, CD8‐Treg ratio in omental tumors. Mean and standard error of the mean are shown. *P < .05

Accumulation of specific regulatory T cell (Treg) populations in ovarian cancer and surrounding ascites is a substantial barrier to the generation of productive anti‐tumor immunity.^20^, ^21^ Because class I HDAC inhibition has been reported to impair Treg programs^22^ and greater CD8‐Treg ratios are associated with improved outcomes in ovarian cancer patients,^12^ we next asked if ENT could alleviate Treg suppression in the ovarian cancer environment. ID8 tumor cells were injected into the peritoneum of C57BL/6 mice, daily vehicle or ENT treatment initiated 21 days thereafter, and tumor‐bearing omentum harvested after 10 days of treatment. Although we found no differences in omental mass (Figure 2A), the CD8‐Treg ratio within ascites fluid was significantly increased (Figure 2B) with a trend toward increased ratio in the corresponding TME (Figure 2C). These data suggest that, in addition to changes in tumor expression profiles, ENT treatment may compromise suppressive networks within the TME and ascites to facilitate CD8 T cell accumulation at the site of disease.

**FIGURE 3:**
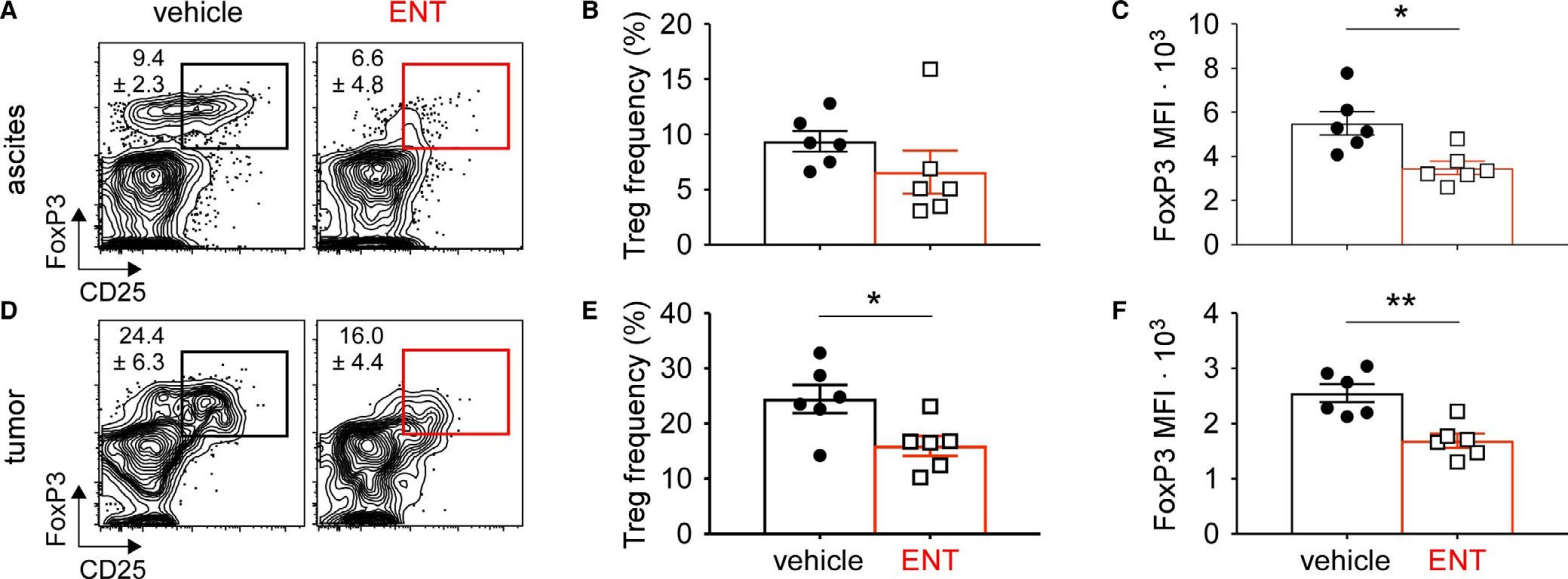
Tumor associated Treg suppression is impaired by ENT. ID8 cells were injected into the peritoneal cavity of C57BL/6 mice, daily vehicle or ENT treatment started on day 21, and omental tumors harvested on day 31. Tregs in tumor and associated ascites were assessed by intracellular staining and flow cytometry. A. Representative flow plots of Tregs in ascites of vehicle and ENT treated mice, gated on live/dead, CD3+, CD4+ cells. Numbers indicate mean ± SD. B, Frequency of Tregs in ascites fluid. C, Per cell expression of FoxP3 measured by MFI in ascites. D, Representative flow plots of intratumoral Tregs, gated on live/dead, CD3+, CD4+ cells. E, Frequency of intratumoral Tregs. F, Per cell expression of FoxP3 measured by MFI in the tumor. Mean and standard error of the mean are shown. *P < .05. **P < .005

In addition to improving the CD8‐Treg ratio, we next asked if class I HDAC inhibition impaired the suppressive capacity of Tregs within the TME and ascites. Using flow cytometry, ENT treatment appeared to reduce the frequency of CD4+ CD25+ FoxP3+ Tregs within the ascites, though this did not reach statistical significance (Figure 3A,B). However, the per cell expression of the Treg lineage‐defining transcription factor FoxP3, measured by mean fluorescent intensity (MFI), experienced a significant decrement following ENT treatment (Figure 3C), suggesting programmatic differences among Tregs following HDAC inhibition. Similarly, the frequency of CD4+ CD25+ FoxP3+ Tregs was significantly reduced in the omentum of ENT‐treated mice (Figure 3D,E) and these cells also possessed a markedly reduced FoxP3 MFI (Figure 3F). Levels of FoxP3 expression directly correlate with Treg suppressive capacity^23^; therefore, these data suggest class I HDAC inhibition impairs the suppressive nature of Tregs in ovarian cancer by altering both their frequency and canonical expression programs.

### 3.3 ENT treatment induces a transcription factor profile consistent with a less dysfunctional state in CD8 T cells

**FIGURE 4:**
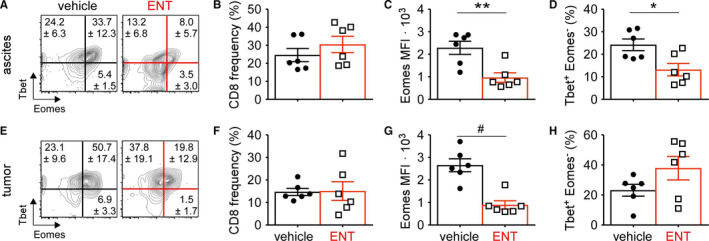
CD8 T cell quality is improved following class I HDAC inhibition. ID8 tumor‐bearing mice were treated daily with vehicle or ENT starting on day 21 and omental tumors and ascites harvested on day 31. Intracellular staining and flow cytometry was used to assess transcription factor expression levels in CD8 T cells. A, Representative flow plots of Tbet vs Eomes expression in CD8 T cells from ascites fluid, gated on live/dead, CD3+, CD8+ cells. Numbers indicate mean ± SD. B, Frequency of CD8 T cells in ascites. C, Per CD8 T cell expression of Eomes measured by MFI in ascites. D, Frequency of Tbet + Eomes‐ CD8 T cells in ascites fluid. E, Representative flow plots of Tbet vs Eomes expression in CD8 T cells from omental tumors, gated on live/dead, CD3+, CD8+ cells. F, Frequency of CD8 T cells in tumors. Numbers indicate mean ± SD. G, Per CD8 T cell expression of Eomes measured by MFI in tumors. H, Frequency of Tbet + Eomes‐ CD8 T cells in the tumor mass. Mean and standard error of the mean are shown. *P < .05. **P < .005. ^#^ *P* < .0005

Although both are required for proper differentiation of effector CD8 T cells, the relative expression levels of Tbet and Eomes transcription factors correlate with exhaustion. Specifically, high levels of Eomes expression predispose CD8 T cells toward declining functionality and an exhausted phenotype.^24^, ^25^ To assess the expression of these transcription factors in responding T cells, ID8 tumor cells were injected into the peritoneal cavity, treatment with vehicle or ENT initiated 21 days later, and CD8 T cells assessed by flow cytometry after 10 days of treatment. Representative flow plots of Tbet vs Eomes show increased expression of the latter in vehicle‐treated ascites samples (Figure 4A). Frequencies of CD8 T cells following vehicle or ENT treatment were not significantly different in ascites (Figure 4B); however, per cell expression of Eomes was substantially higher in vehicle treated mice (Figure 4C). Unexpectedly, fewer CD8 T cells in the ascites exhibited a more effector‐like Tbet + Eomes‐ profile following ENT treatment (Figure 4D). In contrast, intratumoral CD8 T cells were more preferentially Tbet single‐positive following ENT treatment, while vehicle treated samples predominantly expressed Eomes (Figure 4E). Again, we observed no difference in CD8 frequencies (Figure 4F) but Eomes expression was markedly increased in control samples (Figure 4G). CD8 T cells isolated from omental tumors following ENT were more often Tbet‐single positive, though this difference did not reach significance (Figure 4H). Collectively, these findings suggest that class I HDAC inhibition might promote a less terminally differentiated CD8 T cell state.

## 4 DISCUSSION

Immunotherapy is a promising approach to ovarian cancer, as adaptive immunity can reach remote micro‐ and macrometastatic sites and in this way prevent re‐growth of small tumor deposits. In the present study, we have shown that treatment of a spontaneous murine EOC model (MISIIR) with a class I HDAC inhibitor upregulates tumor cell antigen expression and improves CD8 T cell function, while also reducing expression of suppressive myeloid chemoattractants. Next, we used a more rapid, nonspontaneous model (ID8) to demonstrate that these changes in gene expression were associated with an increased CD8‐Treg ratio and compromised Treg suppression program. Finally, CD8 T cells recruited to ID8 tumors following class I HDAC inhibition adopted more favorable transcription factor profiles relative to vehicle treated counterparts.

NanoString analysis of whole tumor lysates and subsequent pathway analysis revealed an upregulation of T cell activation pathways as well as genes in the major histocompatibility class II (MHCII) antigen presentation pathway. Notably, the expression of MHC pathway genes is regulated by interferon gamma (IFNγ) signaling.^26^, ^27^ Increased MHCII pathway expression, then, likely reflects ENT‐induced changes in tumor expression profiles as well as changes in tumor cell biology that in turn promote CD8 T cell activation, subsequent increases in IFNγ production, and resultant upregulation of antigen presentation. In this way, MHC expression reflects a more inflamed TME and may be a biomarker of responsiveness to immunotherapy.^28^

We found ENT increased expression of chemokines driving T cell migration and increase of Tbet single‐positive CD8 T cells in tumors. These changes may indicate an improved ability of Tbet single‐positive CD8 T cells to traffic into or persist in the tumor mass following class I HDAC inhibition. Indeed, class I HDAC inhibition can augment accumulation of IFNγ‐producing CD8 T cells within solid tumors.^29^ A finding consistent with increasing numbers of Tbet‐positive CD8 T cells within tumors following class I HDAC inhibition, as Tbet regulates IFNγ production.^30^

EOC is marked by early and predictable metastasis to the omentum wherein a highly immunosuppressive TME is established, owing to concerted influences of diverse stromal populations and cytokine milieu.^31^ A principal contributor is accumulation of robustly activated Tregs,^32^ including a distinct visceral adipose tissue associated population.^33^ Our data suggest that class I HDAC inhibition can compromise such suppressive networks within the EOC TME to de‐repress cytotoxic functions of infiltrating CD8 T cells. Specifically, ENT treatment reduces the frequency of Tregs within the omental tumor mass and ascites, while also downregulating expression of the lineage‐defining transcription factor FoxP3, consistent with reports in other models.^22^ Because levels of FoxP3 correlate with Treg suppressive capacity,^23^ this suggests that class I HDAC inhibition impairs Treg accumulation and per cell suppression. ENT also increased the CD8‐Treg ratio, though the value was consistently lower in tumors. Higher numbers of Tregs (due to accumulation and potential proliferation) in the omentum at baseline may account for this discrepancy.^20^

Class I HDAC inhibition has also been shown to deplete myeloid derived suppressor cells (MDSCs), another population within the TME delimiting anti‐tumor T and NK cell functions.^34^, ^35^ MDSCs are recruited to the TME by way of CXCR2 ligands CXCL1, CXCL2, CXCL3, and CXCL5,^36^ which we found to be downregulated in the spontaneous MISIIR ovarian tumors following class I HDAC inhibition, suggesting ENT might impair accumulation of MDSCs. Further experimentation is needed, however, to corroborate these findings at the protein level and determine the extent to which ENT functionally impairs MDSC accumulation in ovarian cancer. Collectively, our data suggests that class I HDAC inhibition destabilizes suppressive networks within ovarian tumors, shifting the TME toward one more favorable for anti‐tumor immunity. Additional interventions providing a “second hit” to these suppressive populations or blocking inhibitory signaling could further promote CD8 T cell responses and unleash the potential of immunotherapy in this hostile environment.^29^, ^37^

We observed improvements in CD8 T cell and tumor cell biology but unexpectedly no difference in omental tumor mass. This discrepancy in outcome may be explained by the dosing regimen used, starting treatment after 10 weeks in the MISIIR model or after 3 weeks in the more aggressive ID8 model. Once tumors become established, tumor‐specific T cells begin acquiring a progressively dysfunctional phenotype and, depending on the specific model used, can rapidly become functionally exhausted.^38^ HDAC inhibition is unlikely to restore function in exhausted CD8 T cells; accordingly, these agents may be best implemented as adjuvants in conjunction with other therapies stimulating productive immune responses. Since the timing of class I HDAC inhibition relative to T cell functional status significantly impacts the overall outcome,^16^ clinical implementation will necessitate careful investigation of optimal dose and administration schedule.

In sum, we have demonstrated that class I HDAC inhibition improves activation status of CD8 T cells within the EOC TME, while also disrupting immune suppressive networks. Although class I HDAC inhibitors have thus far failed to demonstrate notable efficacy against solid tumors as single agents,^39^ they may be opportune adjuvants in treatment of EOC. Specifically, taxane and platinum‐based chemotherapeutics stimulate tumor‐specific CD8 T cell responses,^40^, ^41^ which class I HDAC inhibition might elaborate by improving tumor‐specific T cell function and impairing intratumoral suppressive networks. In this sense, appropriate integration of class I HDAC inhibition into treatment regimens may sensitize nonresponsive EOC to checkpoint blockade, availing the success of immunotherapy to this patient population.

## CONFLICT OF INTEREST

The authors have no relevant conflicts of interest to disclose.

## AUTHOR CONTRIBUTIONS

Tyler R. McCaw contributed to conceptualization, data curation, formal analysis, investigation, methodology, supervision, validation, visualization, roles/writing—original draft, writing—review and editing. Nidhi Goel contributed to data curation, formal analysis, investigation, writing—review and editing. Dewey J. Brooke contributed to data curation, formal analysis, methodology, software, validation, visualization, writing—review and editing. Ashwini A. Katre contributed to data curation, investigation, project administration, writing—review and editing. Angelina I. Londoño contributed to data curation, project administration, writing—review and editing. Haller J. Smith contributed to conceptualization, writing—review and editing. Troy D. Randall contributed to conceptualization, data curation, resources, writing—review and editing. Rebecca C. Arend contributed to conceptualization, formal analysis, funding acquisition, investigation, methodology, supervision, validation, project administration, resources, rwiting ‐ review and editing.

## Supporting information

Table S1

Supplementary Material

## Data Availability

Data will be made available upon request.
