# Supplementary material for: Class I histone deacetylase inhibition promotes CD8 T cell activation in ovarian cancer
Source: Cancer Med. 2020 Dec 24;10(2):709–17. doi: 10.1002/cam4.3337 (PMC7877343; doi:10.1002/cam4.3337)
Supplement: Supplementary file 2 — Supplementary Material [file CAM4-10-709-s002.docx]

**Supplemental Table 1.** Top 20 upregulated genes in MISIIR omental tumors following ENT treatment with a brief description of function. Fold change at 10 weeks is shown. Supplemental references detailing potential gene function are provided (1-23).

**Supplemental References:**

1. Lavorgna A, Matsuoka M, Harhaj EW. A critical role for IL-17RB signaling in HTLV-1 tax-induced NF-κB activation and T-cell transformation. PLoS Pathog. 2014;10(10):e1004418.

2. Hofmann M, Pircher H. E-cadherin promotes accumulation of a unique memory CD8 T-cell population in murine salivary glands. Proceedings of the National Academy of Sciences. 2011;108(40):16741-6.

3. Mackay LK, Rahimpour A, Ma JZ, Collins N, Stock AT, Hafon M-L, et al. The developmental pathway for CD103+ CD8+ tissue-resident memory T cells of skin. Nature immunology. 2013;14(12):1294-301.

4. Merindol N, Riquet A, Szablewski V, Eliaou J-F, Puisieux A, Bonnefoy N. The emerging role of Twist proteins in hematopoietic cells and hematological malignancies. Blood cancer journal. 2014;4(4):e206-e.

5. Wu Z, Zhang Z, Lei Z, Lei P. CD14: Biology and role in the pathogenesis of disease. Cytokine & growth factor reviews. 2019;48:24-31.

6. Mathews DV, Dong Y, Higginbotham LB, Kim SC, Breeden CP, Stobert EA, et al. CD122 signaling in CD8+ memory T cells drives costimulation-independent rejection. The Journal of clinical investigation. 2018;128(10):4557-72.

7. Dankner M, Gray-Owen SD, Huang Y-H, Blumberg RS, Beauchemin N. CEACAM1 as a multi-purpose target for cancer immunotherapy. Oncoimmunology. 2017;6(7):e1328336-e.

8. Khairnar V, Duhan V, Patil AM, Zhou F, Bhat H, Thoens C, et al. CEACAM1 promotes CD8+ T cell responses and improves control of a chronic viral infection. Nature communications. 2018;9(1):1-14.

9. Lin L, Spoor MS, Gerth AJ, Brody SL, Peng SL. Modulation of Th1 activation and inflammation by the NF-κB repressor Foxj1. Science. 2004;303(5660):1017-20.

10. Kastenmüller W, Brandes M, Wang Z, Herz J, Egen JG, Germain RN. Peripheral prepositioning and local CXCL9 chemokine-mediated guidance orchestrate rapid memory CD8+ T cell responses in the lymph node. Immunity. 2013;38(3):502-13.

11. Lieber S, Reinartz S, Raifer H, Finkernagel F, Dreyer T, Bronger H, et al. Prognosis of ovarian cancer is associated with effector memory CD8+ T cell accumulation in ascites, CXCL9 levels and activation-triggered signal transduction in T cells. Oncoimmunology. 2018;7(5):e1424672.

12. Floderer M, Prchal-Murphy M, Vizzardelli C. Dendritic Cell-Secreted Lipocalin2 Induces CD8+ T-Cell Apoptosis, Contributes to T-Cell Priming and Leads to a TH 1 Phenotype. PLoS One. 2014;9(7):e101881.

13. Rodvold JJ, Mahadevan NR, Zanetti M. Lipocalin 2 in cancer: when good immunity goes bad. Cancer letters. 2012;316(2):132-8.

14. Pittet MJ, Speiser DE, Valmori D, Cerottini J-C, Romero P. Cutting edge: cytolytic effector function in human circulating CD8+ T cells closely correlates with CD56 surface expression. The Journal of Immunology. 2000;164(3):1148-52.

15. Sohn SJ, Forbush KA, Nguyen N, Witthuhn B, Nosaka T, Ihle JN, et al. Requirement for Jak3 in mature T cells: its role in regulation of T cell homeostasis. The Journal of Immunology. 1998;160(5):2130-8.

16. Deng J, Wang L, Chen H, Li L, Ma Y, Ni J, et al. The role of tumour-associated MUC1 in epithelial ovarian cancer metastasis and progression. Cancer and Metastasis Reviews. 2013;32(3-4):535-51.

17. Cho MS, Rupaimoole R, Choi H-J, Noh K, Chen J, Hu Q, et al. Complement component 3 is regulated by TWIST1 and mediates epithelial–mesenchymal transition. The Journal of Immunology. 2016;196(3):1412-8.

18. Stoeckle C, Gouttefangeas C, Hammer M, Weber E, Melms A, Tolosa E. Cathepsin W expressed exclusively in CD8+ T cells and NK cells, is secreted during target cell killing but is not essential for cytotoxicity in human CTLs. Experimental hematology. 2009;37(2):266-75.

19. Wang JP, Hielscher A. Fibronectin: how its aberrant expression in tumors may improve therapeutic targeting. Journal of Cancer. 2017;8(4):674.

20. Gao GF, Jakobsen BK. Molecular interactions of coreceptor CD8 and MHC class I: the molecular basis for functional coordination with the T-cell receptor. Immunology today. 2000;21(12):630-6.

21. Pedraza‐Alva G, Rosenstein Y. CD43–One molecule, many tales to recount. Signal Transduction. 2007;7(5‐6):372-85.

22. Markowitz GJ, Yang P, Fu J, Michelotti GA, Chen R, Sui J, et al. Inflammation-dependent IL18 signaling restricts hepatocellular carcinoma growth by enhancing the accumulation and activity of tumor-infiltrating lymphocytes. Cancer research. 2016;76(8):2394-405.

23. Chanakira A, Westmark PR, Ong IM, Sheehan JP. Tissue factor-factor VIIa complex triggers protease activated receptor 2-dependent growth factor release and migration in ovarian cancer. Gynecologic oncology. 2017;145(1):167-75.
